# Altered Dendritic Morphology of Purkinje cells in *Dyt1* ΔGAG Knock-In and Purkinje Cell-Specific *Dyt1* Conditional Knockout Mice

**DOI:** 10.1371/journal.pone.0018357

**Published:** 2011-03-29

**Authors:** Lin Zhang, Fumiaki Yokoi, Yuan-Hu Jin, Mark P. DeAndrade, Kenji Hashimoto, David G. Standaert, Yuqing Li

**Affiliations:** 1 Center for Neurodegeneration and Experimental Therapeutics, Department of Neurology, School of Medicine, University of Alabama at Birmingham, Birmingham, Alabama, United States of America; 2 Division of Clinical Neuroscience, Chiba University Center for Forensic Mental Health, Chiba, Japan; Rikagaku Kenkyūsho Brain Science Institute, Japan

## Abstract

**Background:**

DYT1 early-onset generalized dystonia is a neurological movement disorder characterized by involuntary muscle contractions. It is caused by a trinucleotide deletion of a GAG (ΔGAG) in the *DYT1* (*TOR1A*) gene encoding torsinA; the mouse homolog of this gene is *Dyt1* (*Tor1a*). Although structural and functional alterations in the cerebellum have been reported in DYT1 dystonia, neuronal morphology has not been examined *in vivo*.

**Methodology/Principal Findings:**

In this study, we examined the morphology of the cerebellum in *Dyt1* ΔGAG knock-in (KI) mice. Golgi staining of the cerebellum revealed a reduction in the length of primary dendrites and a decrease in the number of spines on the distal dendrites of Purkinje cells. To determine if this phenomenon was cell autonomous and mediated by a loss of torsinA function in Purkinje cells, we created a knockout of the *Dyt1* gene only in Purkinje cells of mice. We found the Purkinje-cell specific *Dyt1* conditional knockout (*Dyt1* pKO) mice have similar alterations in Purkinje cell morphology, with shortened primary dendrites and decreased spines on the distal dendrites.

**Conclusion/Significance:**

These results suggest that the torsinA is important for the proper development of the cerebellum and a loss of this function in the Purkinje cells results in an alteration in dendritic structure.

## Introduction

Dystonia is a neurological syndrome characterized by involuntary contractions of both agonist and antagonist muscles of affected regions that cause twisting and abnormal movements or postures [1]. DYT1 dystonia is a genetically determined form of generalized early-onset dystonia, with an age of onset between childhood and adolescence. Symptoms usually first affect the lower limbs and eventually progress to the entire body [2]. DYT1 dystonia is caused by a trinucleotide deletion of a GAG (ΔGAG) codon in the *DYT1* (*TOR1A*) gene, which results in the loss of a glutamic acid residue in the C-terminal region of the torsinA protein [3]. We previously generated *Dyt1* ΔGAG knock-in (KI) mice, a mouse model of DYT1 dystonia, showed impairments of motor coordination and balance in the beam-walking test and hyperactivity in the open-field test [4]. The function of torsinA is largely unknown, but it is a member of the AAA+ ATPase superfamily and is believed to have a chaperone-like function [5], [6], [7], [8], [9], [10].

Biochemical and cellular studies show that torsinA localizes to the endoplasmic reticulum [6], protects against oxidative stress, and prevents protein aggregate formation [5], [7], [8], [9]. *In situ* hybridization studies have also revealed that torsinA mRNA is highly expressed in the dopaminergic neurons of the substantia nigra pars compacta, granule and pyramidal neurons of the hippocampus, Purkinje and dentate nucleus neurons of the cerebellum, and cholinergic neurons of the neostriatum in humans [11], [12]. Furthermore, ultrastructural studies of the striatum of humans and macaques have revealed an association of torsinA immunostaining with small vesicles within axons and presynaptic terminals forming symmetric synapses [13].

Growing evidence suggests that the structural and/or functional abnormalities in the cerebellum could be involved in the pathogenesis of dystonia. Brain imaging studies have revealed structural grey matter changes in the cerebellum of patients with upper limb dystonia [14], cervical dystonia, and blepharospasm [15], [16]. Increased activation of the cerebellum in the patients with DYT1 dystonia carriers and alterations in the olivo-cerebellar pathway of patients with primary focal dystonia have been reported [17]. Furthermore, there are several reports showing that trauma to the cerebellum or cerebellar atrophy can cause dystonia [18], [19]. In a genetically dystonic rat that harbors a mutation in the gene *caytaxin*, cerebellectomy eliminates the motor symptoms and rescues the juvenile lethality [20], [21]. Electronic lesions of dorsal portions of the lateral vestibular nuclei (dLV), which receive input from the Purkinje cells, are associated with the greatest improvement in this rat [22]. The Purkinje cells send the major inhibitory signal from the cerebellum to the deep cerebellar nuclei, which is mediated by the neurotransmitter γ-aminobutyric acid (GABA). Pharmacological disruption of the cerebellar signaling is also shown to induce dystonia in mice [23]. Lastly, the tottering mouse, which has a recessive mutation of a calcium channel gene, shows ataxia and paroxysmal dystonia, but this phenotype can be eliminated by surgical removal of the cerebellum or introduction into a Purkinje cell-specific degenerative background [24], [25].

Despite these findings, few studies have sought to examine the potential role of the Purkinje cells in the pathogenesis of DYT1 dystonia and other dystonias. In this study, we examined the morphology of the cerebellum in *Dyt1* ΔGAG knock-in (KI) mice. Golgi staining of the cerebellum of KI mice revealed a reduction in the length of primary large dendrites and a decrease in the number of spines on the distal dendrites of Purkinje cells. Since it has been reported that the ΔGAG mutation causes a reduction of torsinA in the striatum and the entire brain [26], [27], [28], we sought to determine if this phenomenon was mediated by a loss of function of torsinA in Purkinje cells by creating a knockout of the *Dyt1* gene only in Purkinje cells (*Dyt1* pKO) of mice. We previously reported the making of *Dyt1 loxP* mice [29]. In the present study, we produced *Dyt1* pKO mice by crossing the *Dyt1 loxP* mice with *Pcp2-cre* mice, which restricts lox-mediated recombination to Purkinje cells [30]. We found that the Purkinje cells in the *Dyt1* pKO mice have a similar morphology to that of the KI mice, with shortened primary large dendrites and decreased spines on the distal dendrites.

## Results

### Golgi staining of Purkinje cells in KI mice

**Figure 1:**
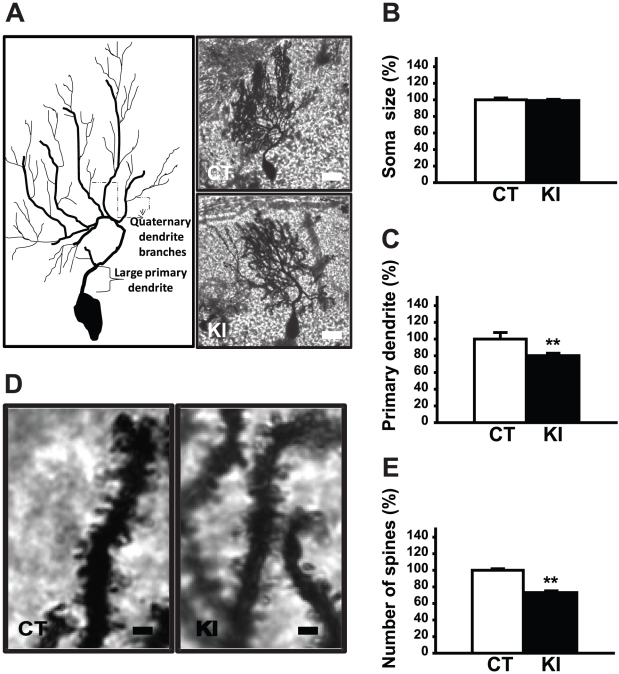
Purkinje cells in KI mice. (A) A representative Purkinje cell trace (left). A representative Purkinje cell from CT and KI mice at 40× magnification (right). (B) There was no significant difference in size of the Purkinje cell soma between CT and KI mice. (C) However, the large primary dendrite of the Purkinje cells in the KI mice was significantly shorter than those in CT mice. (D) Next, the quaternary dendrite branch of CT and KI mice was examined at 100× magnification. (E) The number of spines on the quaternary dendrite branch in the KI mice was significantly reduced compared to CT mice. Scale bars in Panel A represent 10 µm. Scale bars in panel D represent 1 µm. Bars in Panels B, C and E represent means with standard errors. ** p<0.01.

To examine the morphological structures of the Purkinje cells in KI mice, we used Golgi staining of cerebellar sections. First, the sizes of the Purkinje cell soma were measured and no significant difference was found between the KI and control (CT) mice (means ± standard errors; CT: 100±2.22%; KI: 99.19±1.35%; p>0.05, Figure 1A–1B). However, the length of the large primary dendrite in the KI mice was approximately 20% shorter than those in the control mice (CT: 100±7.84%; KI: 80.15±2.95%; p<0.01, Figure 1C). Furthermore, the number of spines in the quaternary dendrite branch of KI mice was approximately 27% percent less than those in control mice (CT: 100±1.9%; KI: 73.11±2.17%; p<0.01, Figure 1D–1E). These results suggest the important role of torsinA in the dendritic structure and morphology of Purkinje cells.

### Generation of the Purkinje-cell specific *Dyt1* conditional knockout (*Dyt1* pKO) mice

**Figure 2:**
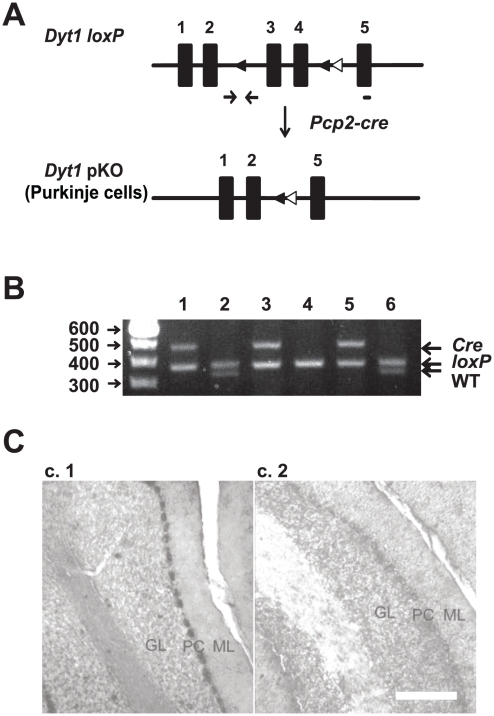
Generation of *Dyt1* pKO mice. (A) Schematic diagram of the generation of the *Dyt1* pKO mice. Filled boxes represent exons. Filled triangles indicate *loxP* sites. Open triangles indicate the *FRT* sites that were incorporated to remove the *neo* cassette. *Dyt1 loxP* mice were crossed with *Pcp2-cre* mice to obtain double heterozygotes. The double heterozygotes were crossed with *Dyt1 loxP* homozygotes to obtain *Dyt1* pKO mice. The primer sites for genotyping of *Dyt1* locus were shown by an arrow pairs. The short bar under exon 5 is the site of probe used for *in situ* hybridization. *Dyt1* exons 3 and 4 were removed in Purkinje cells of *Dyt1* pKO mice. (B) An agarose gel showing the various PCR products that were used to genotype mice. The top band indicates the presence of the *Pcp2-cre* locus, the middle band represents the *Dyt1 loxP* locus, and the bottom band represents the *Dyt1* wild-type locus. Lanes 4: *Dyt1 loxP* homozygous mice. Lanes 2, 6: *Dyt1 loxP* heterozygous mice. Lanes 1, 3, 5: *Dyt1* pKO mice. (C) *In situ* hybridization was used to confirm the Purkinje cell-specific knockout of the *Dyt1* gene. CT (C.1) and *Dyt1* pKO (C.2) mice. GL: granule cell layer; PC: Purkinje cell layer; ML: molecular layer. Scale bar represents 100 µm in C.2.

To examine whether the morphological alterations in the KI mice was caused by a loss of torsinA function in Purkinje cells, we generated a Purkinje cell-specific knockout of the *Dyt1*. *Dyt1 loxP* mice [29] were crossed with a line of mice with the *cre* recombinase gene driven by the promoter of *Pcp2*, a Purkinje cell-specific gene [30]. Mice double heterozygous for both the *Dyt1 loxP* and *Pcp2-cre* were then crossed with *Dyt1 loxP* homozygous mice to derive *Dyt1* pKO mice and their control littermates (Figure 2A). Genotyping for *Dyt1* pKO and control littermates was performed by multiplex PCR (Figure 2B). We confirmed Purkinje cell specific knockout of *Dyt1* by *in situ* hybridization. TorsinA mRNA was highly expressed in the Purkinje cells in control littermates (Figure 2C.1). In contrast, torsinA mRNA was not detected in Purkinje cells in *Dyt1* pKO mice (Figure 2C.2), suggesting *Dyt1* was specifically knocked out in the Purkinje cells.

### Golgi staining of Purkinje cells in *Dyt1* pKO mice

**Figure 3:**
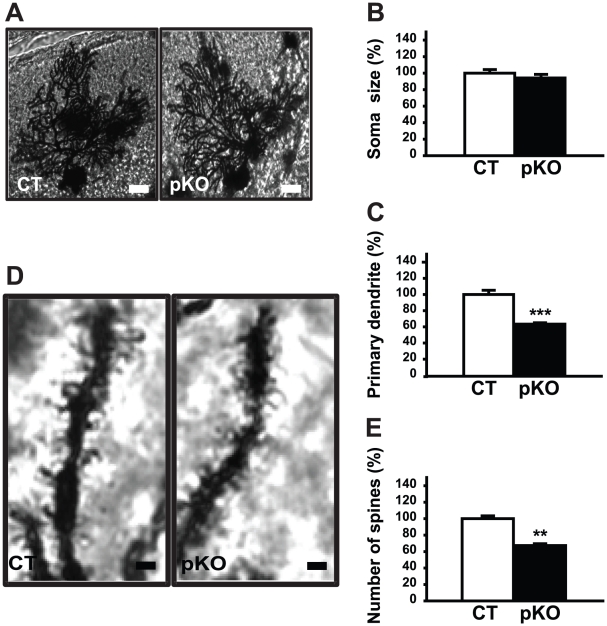
Purkinje cells in *Dyt1* pKO mice. (A) A representative Purkinje cell from control and *Dyt1* pKO mice, produced at 40× magnification. (B) The size of the Purkinje cell soma in *Dyt1* pKO mice was not significantly different than those of CT mice. (C) The large primary dendrite of the Purkinje cells in the *Dyt1* pKO mice was significantly shorter than those in CT mice. (D) A representative quaternary dendrite branch of CT and *Dyt1* pKO mice was examined at 100× magnification. (E) The number of spines on the quaternary dendrite branch in the *Dyt1* pKO mice were significantly reduced compared to those of CT mice. Scale bars in Panel A represent 10 µm. Scale bars in Panel D represent 1 µm. Bars in Panels B, C and E represent means with standard errors. ** p<0.01, *** p<0.001.

To examine whether the *Dyt1* pKO mice recapitulate the dendritic morphology of the KI mice, we performed Golgi staining on cerebellar sections from *Dyt1* pKO mice at approximately 8 months old. Similar to the KI mice, no significant difference in the size of the Purkinje cell soma in *Dyt1* pKO mice compared to control mice was observed (CT: 100±3.20%; *Dyt1* pKO: 94.27±2.82%; p>0.05, Figure 3A–3B). Furthermore, the length of the large primary dendrite in the *Dyt1* pKO mice was approximately 37% shorter than those in control mice (CT: 100±5.65%; *Dyt1* pKO 63.36±1.73%; p<0.001, Figure 3C). Lastly, the number of spines in the quaternary dendrite branch of the *Dyt1* pKO mice was approximately 33% less than those in control mice (CT: 100±3.45%; *Dyt1* pKO: 67.36±2.2%; p<0.01, Figure 3D–3E). These results suggest that torsinA plays an important role in the Purkinje cell dendritic development.

To examine whether the morphological alteration in Purkinje cells was developmentally regulated, we analyzed the Purkinje cells in *Dyt1* pKO mice at 2–3 months old. No significant difference in the size of the Purkinje cell soma in *Dyt1* pKO mice was observed compared to control mice (CT: 100±3.71%; *Dyt1* pKO: 98.01±2.52%; p>0.05). There was also no significant difference in the length of the large primary dendrite in *Dyt1* pKO mice compared to control mice (CT: 100±3.81%; *Dyt1* pKO 90.68±3.37%; p>0.05). However, the number of spines in the quaternary dendrite branch of the Purkinje cells was reduced in *Dyt1* pKO mice compared to control mice (CT: 100±0.55%; *Dyt1* pKO: 93.58±0.85%; p<0.001). The results suggest that the spine numbers reduced in advance of the reduction of the length of the large primary dendrite by the loss of torsinA function in the Purkinje cells.

## Discussion

First, we examined the morphology of Purkinje cells in the cerebellum in KI mice. We found that the primary large dendrites of the Purkinje cells in KI mice were significantly shorter than that of wild type mice and there was a marked reduction in the number of spines. Next, to determine if this morphological change was mediated by a cell-autonomous effect of loss of torsinA function, we generated a line of mice in which torsinA was conditionally knocked out only in Purkinje cells. The *Dyt1* pKO mice showed a similar decrease in the length of primary large dendrites and a reduction in the number of spines. The results also suggest that the spine numbers reduced in advance of the reduction of the length of the large primary dendrite by the loss of torsinA function in the Purkinje cells. These results suggest that torsinA plays an important role in the development of the cerebellum, and that a loss of this function in the Purkinje cells results in a cell autonomous effect leading to an alteration in dendritic structure. If the same alterations are present in patients with DTY1 dystonia, this change in synaptic associations between the parallel fibers and the Purkinje cells may contribute to the pathogenesis of the dystonic symptoms.

Growing evidence suggests that structural and functional abnormalities in the brain could be involved in the pathogenesis of dystonia [14], [15], [16], [31]. However, neuronal morphology of the cerebellum has not been examined *in vivo*. A recent report suggests that DYT1 dystonia is a neurodevelopmental disorder involving the cortico-striatal-pallido-thalamocortical and the cerebellar-thalamo-cortical pathways [32]. Anatomical studies have proposed an interaction between the cerebellum and the basal ganglia through a disynaptic pathway originating in the cerebellum and projecting to the striatum via the thalamus [33]. A deficiency in this connectivity was identified in patients with DYT1 human carriers [34]. Additionally, rats that have undergone a hemicerebellectomy were found to have a complete loss of striatal long-term depression (LTD) [35], which was also compromised in mutant human torsinA transgenic mice [36]. Since the Purkinje cells are the sole output from the cerebellum, the abnormal function of Purkinje cells, as represented by our results, may be responsible for the change in striatal LTD in mutant torsinA animals.

The Purkinje cells play an important role in motor coordination and motor learning by integrating two types of excitatory inputs: climbing fibers and parallel fibers. Climbing fibers originate from the inferior olivary nucleus and convey the motor signals to the parallel fibers. Parallel fibers are the T-shaped axons of cerebellar granule cells, and convey the sensory and motor information carried through the pontocerebellar and spinocerebellar mossy fiber pathways. It is also known that the parallel fiber-Purkinje cell (PF-PC) synapse plays an important role in the adaptive learning process [37]. Glutamate receptor δ2 subunit (GluRδ2) is selectively expressed in the Purkinje cells of the cerebellum [38]. Impairment of motor coordination, Purkinje cell synapse formation, and cerebellar LTD was reported in GluRδ2 mutant mice [39]. GluRδ2 mutant mice were unable to stabilize PF-PC synapses and resulted in a reduction in the number of PF-PC synapses along with impaired CF synapse elimination [40]. We previously reported motor deficits in KI mice [4] and in this study found morphological alterations of Purkinje cells in KI mice. The results suggest that functional alterations of the cerebellum may associate with the pathogenesis of DYT1 dystonia.

Furthermore, torsinA is known to interact with kinesin 1 [41], a motor protein involved in cellular transport and the cytoskeleton of the cell. *In vitro* studies have shown that suppression of kinesin leads to decreased neurite extension in hippocampal neurons [42]. Furthermore, in human neuroblastoma cells, it was shown that overexpression of mutant torsinA also leads to decreased neurite extension [43]. A decrease in neurite extension possibly through kinesin could explain the decrease in primary dendritic length. In addition to the shortened primary dendrite length, however, the KI mice also showed a decrease in spine number. This decrease in neurite extension would not explain the decrease in number of spines. Recent reports have shown that overexpression of mutant human torsinA in *Drosophila* results in altered synaptic morphology at the neuromuscular junction [44]. It is, therefore, likely that this decrease in spine number results in altered synaptic plasticity, possibly leading to decreased parallel fiber connectivity.

Lastly, we have created a novel Purkinje cell-specific knockout of *Dyt1* to compare its morphology to the KI and wild-type mice. Several genetic studies suggest that a loss-of-function of torsinA contributes to the pathology of dystonia [28], [29], [45]. We have found the *Dyt1* pKO mice replicates the KI dendritic morphology of Purkinje cells, in that they have decreased primary dendrite length and decreased spine number. These findings suggest that the ΔGAG mutation in the KI mice results in a loss of function of torsinA and provide further evidence of the important role of torsinA in the cerebellum.

In conclusion, these results add to the growing body of evidence of the importance of the cerebellum in the pathogenesis of dystonia and this being the first reported morphological alteration in the cerebellum. Furthermore, these results suggest that torsinA plays an important role in the regulations of dendritic length and spine number in the cerebellum. Finally, the ΔGAG mutation in the *Dyt1* may result in a loss of function of torsinA in the Purkinje cells. These findings will further the understanding of the pathophysiology that underlies not only DYT1 dystonia but also possibly other neurological movement disorders.

## Materials and Methods

### Animals

All experiments were carried out in compliance with the USPHS Guide for Care and Use of Laboratory Animals and approved by the Institutional Animal Care and Use Committee at the University of Alabama at Birmingham with Animal Protocol Number 10008918. All experiments were performed by investigators blind to the genotype of the mice. *Dyt1 loxP* mice were generated as previously described [29]. To generate the Purkinje cell-specific knockout, *Dyt1 loxP* mice were first crossed with *Pcp2-cre* mice [30]. The double heterozygous mice were then crossed with *Dyt1 loxP* homozygous mice to derive *Dyt1* pKO mice and their control littermates. Genotyping for *Dyt1* pKO and control littermates was performed by multiplex PCR using F (5′-ATTCAAAAATGTTGTCATAGCCAGG-3′) and T (5′-CTACAGTGACCTGAATCATGTGGC-3′) primer sets [29] for *Dyt1 loxP* and creA (5′-ATCTCCGGTATTGAAACTCCAGCGC-3′) and cre6 (5′-CACTCATGGAAAATAGCGATC-3′) primer sets for *cre* [46]. KI mice were prepared and genotyped as previously described [4]. Mice were housed under a 12-h-light/dark cycle with *ad libitum* access to food and water.

### *In Situ* Hybridization

Purkinje-cell specific knockout of torsinA was confirmed by *in situ* hybridization. To prepare the Digoxigenin (DIG) -labeled probe, a DNA fragment corresponding to 3′-UTR of *Dyt1* was amplified by PCR with a primer sets of Dyt1insitu2 (5′-CACCAAGCTGGACTACTACCTGGA-3′) and Dyt1insitu3 (5′-GAAAGCTTCTTATAGTATTAAAACC-3′) and *Dyt1* DNA plasmid template. The amplified PCR fragment was then ligated into a pGEM-T Easy vector (Promega). Next, the construct was transformed in *E. coli* JM109 competent cells (Promega) and single colonies were isolated. An appropriate clone that had the DNA fragment in the correct direction was confirmed by PCR using T7 and Dyt1insitu3 primer sets. The plasmid DNA was purified and cut with the restricted enzyme *Nco*I. The DNA fragment was purified and dissolved in diethyl pyrocarbonate (DEPC)-treated water. DIG-labeled probe for *Dyt1* was prepared by using digoxingenin RNA labeling kit with the SP6 promoter (Roche Applied Science, Indianapolis, IN). *In situ* hybridization to sagittal sections of the cerebellum was performed as previously described [47].

### Golgi staining

Adult KI mice (CT: n = 4; KI: n = 7, approximately 8 months old), *Dyt1* pKO mice (CT: n = 3; *Dyt1* pKO: n = 3, approximately 8 months old), and another batch of *Dyt1* pKO mice (CT: n = 4; *Dyt1* pKO: n = 4, 2–3 months old) were used in this experiment. After mice were deeply anesthetized with pentobarbital (1 ml/kg, intraperitoneally), the cerebellum was quickly removed and prepared for Golgi staining using the FD Rapid Golgi Stain Kit (FD NeuroTechnologies, Ellicot City, MD). After staining, the cerebellum was frozen with dry ice and sectioned parasagittally (150 µm) using a sliding microtome (Histoslide 2000, Reichert-Jung). The sections were then mounted on slide, dehydrated with xylene and then cover-slipped with permount (Fisher Scientific). Images of individual Purkinje cells were captured using a Nikon ECLIPSE E800M microscope with a 40× Plan Fluor objective lens. The size of each Purkinje cell soma and the length of the large primary dendrite (5 to 16 cells from each mouse) were measured using ImageJ software (NIH, Ver. 1.42 g). Quaternary dendrite branches from the soma as shown in Figure 1A were chosen at random and the spines were counted. The numbers of spines located on randomly selected quaternary dendrite branches (5 to 16 cells from each mouse), 10 µm in length, were counted manually using a 40× and 100× Plan Fluor objective lens and a 10× CFIUW ocular lens. The size of Purkinje cell soma, the length of the large primary dendrite, and the spine numbers were measured by an investigator blind to the genotypes and the data were analyzed by another investigator.

### Statistical analysis

The area (µm^2^) of each Purkinje cell soma and the length of the large primary dendrite (µm) were measured and the data were normalized to control mice and expressed as percentage. The number of spines were counted and normalized to that of control mice. All results were analyzed using Student's t-test. Significance was assigned by a *P*-value less than 0.05.
